# Ceftaroline and ceftobiprole monotherapy for the treatment of *Staphylococcus aureus* infections: a systematic review and Bayesian meta-analysis

**DOI:** 10.1093/jac/dkag244

**Published:** 2026-07-14

**Authors:** Emanuele Rando, Eugenia Magrini, Flavio Sangiorgi, Federica Salvati, Francesca Catania, Marta Chiuchiarelli, Elena Matteini, Massimo Fantoni, Carlo Torti, Luis Eduardo López-Cortés, Jesús Rodríguez-Baño, Rita Murri

**Affiliations:** Dipartimento di Sicurezza e Bioetica – Sezione di Malattie Infettive, Università Cattolica del Sacro Cuore, Rome, Italy; Unidad Clínica de Enfermedades Infecciosas y Microbiología, Hospital Universitario Virgen Macarena, Departamento de Medicina, Facultad de Medicina, Universidad de Sevilla, Instituto de Biomedicina de Sevilla/CSIC, Seville, Spain; Centro de Investigación Biomédica en Red de Enfermedades Infecciosas (CIBERINFEC), Madrid, Spain; Dipartimento di Sicurezza e Bioetica – Sezione di Malattie Infettive, Università Cattolica del Sacro Cuore, Rome, Italy; Dipartimento di Sicurezza e Bioetica – Sezione di Malattie Infettive, Università Cattolica del Sacro Cuore, Rome, Italy; National Institute for Infectious Diseases Lazzaro Spallanzani-IRCCS, Rome, Italy; Dipartimento di Sicurezza e Bioetica – Sezione di Malattie Infettive, Università Cattolica del Sacro Cuore, Rome, Italy; Clinic of Infectious Diseases, San Paolo Hospital, ASST Santi Paolo e Carlo, Department of Health Sciences, University of Milan, Milan, Italy; Dipartimento di Sicurezza e Bioetica – Sezione di Malattie Infettive, Università Cattolica del Sacro Cuore, Rome, Italy; National Institute for Infectious Diseases Lazzaro Spallanzani-IRCCS, Rome, Italy; Dipartimento di Scienze Mediche e Chirurgiche, Fondazione Policlinico Universitario A. Gemelli IRCCS, Rome, Italy; National Institute for Infectious Diseases Lazzaro Spallanzani-IRCCS, Rome, Italy; Dipartimento di Scienze Mediche e Chirurgiche, Fondazione Policlinico Universitario A. Gemelli IRCCS, Rome, Italy; Dipartimento di Sicurezza e Bioetica – Sezione di Malattie Infettive, Università Cattolica del Sacro Cuore, Rome, Italy; Dipartimento di Scienze Mediche e Chirurgiche, Fondazione Policlinico Universitario A. Gemelli IRCCS, Rome, Italy; Unidad Clínica de Enfermedades Infecciosas y Microbiología, Hospital Universitario Virgen Macarena, Departamento de Medicina, Facultad de Medicina, Universidad de Sevilla, Instituto de Biomedicina de Sevilla/CSIC, Seville, Spain; Centro de Investigación Biomédica en Red de Enfermedades Infecciosas (CIBERINFEC), Madrid, Spain; Unidad Clínica de Enfermedades Infecciosas y Microbiología, Hospital Universitario Virgen Macarena, Departamento de Medicina, Facultad de Medicina, Universidad de Sevilla, Instituto de Biomedicina de Sevilla/CSIC, Seville, Spain; Centro de Investigación Biomédica en Red de Enfermedades Infecciosas (CIBERINFEC), Madrid, Spain; Dipartimento di Sicurezza e Bioetica – Sezione di Malattie Infettive, Università Cattolica del Sacro Cuore, Rome, Italy; Dipartimento di Scienze Mediche e Chirurgiche, Fondazione Policlinico Universitario A. Gemelli IRCCS, Rome, Italy

## Abstract

**Background:**

The comparative efficacy of ceftaroline and ceftobiprole against standard of care for *Staphylococcus aureus* infections, including methicillin-resistant *S. aureus* remains uncertain. Thus, we conducted a systematic review and Bayesian meta-analysis to assess the efficacy and safety of ceftaroline and ceftobiprole monotherapy for non-urinary *S. aureus* infections.

**Methods:**

We registered the protocol in PROSPERO. We searched PubMed, Embase, Scopus, and Web of Science. We included randomized controlled trials and comparative non-randomized studies reporting adjusted estimates, enrolling patients with non-urinary *S. aureus* infections. The primary analysis assessed clinical cure in randomized trials using a Bayesian random-effects model with a vague prior. For ceftaroline, we additionally conducted an exploratory analysis using an observationally informed prior derived from non-randomized studies with variance inflation. Risk of bias was assessed with RoB 2 and ROBINS-I and certainty of evidence with GRADE.

**Results:**

Sixteen studies met inclusion criteria [11 ceftaroline (1030 subjects); 5 ceftobiprole (1265 subjects)]. In the primary analysis, the pooled odds ratio for clinical cure was 1.38 (95% CrI 0.81–2.44) for ceftaroline and 0.99 (95% CrI 0.59–1.62) for ceftobiprole. The exploratory ceftaroline analysis using an observationally informed prior yielded an odds ratio of 1.45 (95% CrI 0.91–2.38). Adverse events and serious adverse events were similar between groups. Certainty of evidence was low for ceftaroline and moderate for ceftobiprole.

**Conclusions:**

Current evidence does not demonstrate superiority of ceftaroline or ceftobiprole over standard-of-care therapy for clinical cure in non-urinary *S. aureus* infections. Estimates remain imprecise and are largely driven by registrational trials and subgroup data. These findings support cautious, indication-specific interpretation rather than routine preference of these agents over established therapies.

## Introduction

*Staphylococcus aureus* is responsible for a broad spectrum of clinical syndromes.^1^ Despite advances in supportive care and infection management, *S. aureus* infections continue to be associated with substantial mortality.^1^ Although optimal management requires a multifaceted approach, appropriate antibiotic therapy remains the cornerstone of treatment.^1^ However, several therapeutic uncertainties persist. Questions remain regarding the comparative effectiveness of available agents and whether newer antimicrobials may offer clinical advantages over established therapies.^2^ Over the past two decades, only a limited number of novel conventional antibiotics with activity against *S. aureus* have been developed. Among these, the cephalosporins ceftaroline and ceftobiprole have attracted particular interest.^3,4^ As β-lactam agents, they combine activity against methicillin-resistant *S. aureus* with the pharmacodynamic properties and safety profile traditionally associated with this class.^4^ Their relevance is especially notable in MRSA infections, which have been associated with higher mortality compared with methicillin-susceptible *S. aureus*, potentially influenced by multiple factors, including reliance on non-β-lactam therapies such as vancomycin.^5^

Evidence supporting the use of ceftaroline and ceftobiprole in *S. aureus* infections derives from heterogeneous sources. With the exception of a recent randomized controlled trial specifically conducted in patients with *S. aureus* bacteraemia treated with ceftobiprole,^3^ registrational trials were performed in patients with pneumonia or skin and skin structure infections irrespective of microbiological aetiology. Registrational trials are designed to establish efficacy and safety under controlled conditions and predefined indications, which may limit the direct applicability of their findings to other clinical scenarios.^6^ In parallel, numerous observational studies have explored the off-label use of these agents. However, many clinically relevant uses of these agents, particularly for invasive *S. aureus* infections, are supported mainly by non-randomized studies. A synthesis focused specifically on *S. aureus* infections is therefore needed to clarify the extent to which available data support these agents compared with standard-of-care therapy, while distinguishing between randomized and non-randomized evidence and explicitly accounting for uncertainty.^7,8^ Accordingly, we conducted a systematic review and Bayesian meta-analysis to evaluate ceftaroline and ceftobiprole monotherapy compared with standard-of-care anti-staphylococcal therapy for clinical cure in patients with non-urinary *S. aureus* infections.

## Methods

We registered the protocol of this systematic review on PROSPERO on 11 November 2023 with the number CRD42023478127. We followed the Preferred Reporting Items for Systematic Reviews and Meta-analysis (PRISMA) checklist (Supplemental Material, available as Supplementary data at *JAC* Online). We used Covidence systematic review software (Veritas Health Innovation, Melbourne, Australia, available at www.covidence.org) for article screening, full-text review, and data extraction. We performed the quality assessment of the studies using independent text files.

### Library source and search strategy

To search the evidence systematically, we used the PubMed, Embase, Scopus, and Web of Science databases. We reported the search string in Table S1. We did not use any filter, language or date restrictions. The first search was launched on 2 November 2023. Considering the total duration of the study, we launched a second search with the same string on 26 May 2025 to account for new relevant publications.

### Eligibility criteria

We applied the following inclusion criteria: (i) randomized controlled trials or comparative non-randomized studies of interventions; for non-randomized studies, adjusted estimates for the outcome of interest were required; (ii) studies focusing on *S. aureus* infections in any body site except the urinary tract in adults; (iii) studies comparing ceftaroline or ceftobiprole monotherapy with any other drug with *in vitro* activity against *S. aureus*; (iv) providing data on all-cause mortality at 30 (±2) days when available or clinical cure according to authors’ definition; (v) articles published in journals with peer-review process. No language restriction applied. Monotherapy was defined as the use of ceftaroline or ceftobiprole as the only agent with intended anti-staphylococcal activity in the intervention arm. Combination therapy was permitted provided that no more than one agent with *in vitro* activity against *S. aureus* was used in the intervention arm only; additional agents lacking anti-staphylococcal activity were allowed in both arms when required by the syndrome. We excluded case reports and series, animal or *in vitro* studies, narrative or systematic reviews, as well as meta-analyses and guidelines. We also excluded study protocols, conference papers, preprints, and abstracts. The exclusion of urinary tract infections was intended to avoid including a rare and clinically distinct syndrome in which *S. aureus* isolation may reflect different underlying mechanisms, including haematogenous seeding or colonization, rather than a typical primary urinary infection.

### Screening, full-text review, and data extraction

After removing duplicates, two independent and blind reviewers (F. S. and E. M.) screened the articles based on title and abstract. A third author (F. C.) resolved any disagreement. Subsequently, two authors (F. S. and M. C.) performed the full-text revision independently and blindly. A third blind author (F. C.) resolved any disagreements. Two authors (E. R. and E. M.) prepared the data extraction sheets for randomized trials and non-randomized studies. These contained information on authors, country where the study was conducted, year of publication, study design, intervention, comparator, the adjustment variables in case of non-randomized studies, number of total *S. aureus* isolates as well as methicillin-susceptible and resistant isolates, clinical cure definitions, and data on adverse events and serious adverse events. Then, two independent authors extracted data from randomized trials (F. S. and E. M.), and non-randomized studies (F. Sangiorgi and F. Salvati). A third author resolved any conflicts, consulting with the authors in case clarifications were needed (E. R.). Covidence was used to manage the review workflow.

### Bias assessment

We used the ROBINS-I tool to assess the risk of bias for non-randomized studies of interventions, and the RoB 2 tool for randomized controlled trials. Two authors applied those tools to each study independently (E. M. and F. S. for non-randomized studies, and F. Sangiorgi and F. Salvati for randomized controlled trials). A third blind author resolved conflicts when present, consulting with the authors when appropriate (E. R. for non-randomized studies, and E. M. for randomized controlled trials). The risk-of-bias assessment was performed in separate Excel and Word documents.

### Data synthesis

When we conceived the study, we did not plan to perform a quantitative synthesis of data because of our prior expectations regarding the number of available studies and the quality of their reporting. However, once we completed the full-text screening phase, we amended the protocol on PROSPERO to establish a data analysis plan before inspecting the final dataset. This amendment was made before conducting the quantitative analyses of treatment effects.

Clinical cure and all-cause mortality were prespecified as main outcomes in the PROSPERO registration. However, only clinical cure was sufficiently and consistently reported across *S. aureus* trial subgroups to allow quantitative synthesis. Mortality data were not consistently available for *S. aureus* subgroups; therefore, mortality was not meta-analysed. The primary quantitative analysis consisted of a Bayesian random-effects meta-analysis restricted to randomized controlled trials, using a vague prior for the overall treatment effect. For this analysis, we extracted data from the *S. aureus* subgroups of controlled trials whenever the study did not exclusively recruit participants with *S. aureus* infection. A random-effects model was selected *a priori* because the included trials differed in infection syndrome, study population, comparator regimen, and clinical cure assessment, making a single common-effect assumption clinically implausible. We then performed a random-effects Bayesian meta-analysis of the randomized studies using a vague informative prior, specified as a normal distribution for log odds with a mean of 0 and a standard deviation of 2. We calculated the pooled effect, and the *I*^2^ statistic for heterogeneity to facilitate interpretation of heterogeneity, while recognizing its limited reliability when few studies were available. We specified a half-normal prior for *τ* (0, 0.5). Posterior summaries for *τ* were obtained from the model output.

For ceftaroline, we additionally conducted an exploratory sensitivity analysis using prior information derived from non-randomized studies. Adjusted odds ratios and corresponding 95% confidence intervals from non-randomized studies were converted to the log odds ratio scale with corresponding standard errors. A separate Bayesian random-effects meta-analysis of these estimates was then performed. The resulting posterior distribution for the summary effect was used to construct an observationally informed prior for the RCT analysis. Because most non-randomized studies were judged to be at serious or critical risk of bias, this analysis was considered exploratory. To discount the influence of non-randomized evidence, we inflated the standard error of the observationally derived prior by multiplying by 1.5 the standard error. To assess the influence of this choice, we repeated the analysis using more conservative discounting factors, inflating the standard error by factors of 2 and 3. The planned sensitivity analysis restricted to non-randomized studies at low or moderate risk of bias could not be performed as originally specified because only one non-randomized study was judged to be at moderate risk of bias and no study was judged to be at low risk of bias. We considered that deriving a meta-analytic informative prior from a single non-randomized study would be unstable and potentially misleading.

Subgroup analyses were considered exploratory. The prespecified subgroup analysis examined MRSA infections within randomized trials. An additional exploratory subgroup analysis stratified studies by infection syndrome, separating acute bacterial skin and skin structure infections from pneumonia and bacteraemia when data allowed. Given the small number of studies contributing to each subgroup, these analyses were interpreted descriptively and were not used to draw definitive syndrome-specific conclusions.

As additional outcomes, we estimated the pooled effect for the incidence of adverse events and serious adverse events across the overall trial populations using a random-effects Bayesian meta-analysis. For this analysis, we only applied a vague informative prior. We conducted all analyses separately for ceftaroline and ceftobiprole. Because safety data were generally reported for the overall trial populations rather than *S. aureus*-specific subgroups, these analyses were interpreted as drug-level safety summaries rather than syndrome-specific safety estimates. To assess the robustness of findings, we performed a sensitivity analysis by repeating all the analyses under a frequentist approach.

Finally, we evaluated the certainty of evidence for the efficacy of ceftaroline and ceftobiprole monotherapy for *S. aureus* infections using the Grading of Recommendations, Assessment, Development, and Evaluations (GRADE) methodology. Certainty of evidence was assessed independently by two reviewers (E. R. and J. R. B.), with disagreements resolved by discussion or consultation with a third reviewer.

We performed the statistical analyses with R software v4.2.2 and RStudio v2023.06.0 + 421 https://www.R-project.org/ [accessed on 24 September 2025]. The main packages used for data synthesis and graphical/statistical outputs were brms, meta, metafor, and tidybayes.

## Results

Initially, we ran a first search on 2 November 2023 finding 6574 articles. Considering that the study took more than one year from that date to be completed, we updated the review with a new search on 26 May 2025, adding 436 studies. Overall, our search found 7010 articles, of which 3538 duplicates were removed. We screened 3472 articles, 99 underwent a full-text review, finally retrieving 16 studies to be included in the review. The full PRISMA diagram is reported in Figure S1. All articles were in English.

### Characteristics of the studies

We retrieved 16 studies in total, 11 evaluating ceftaroline^9–19^ and 5 evaluating ceftobiprole.^3,20–23^ All ceftobiprole studies were randomized controlled trials, whereas among the ceftaroline studies, six were randomized^14–19^ and the remainder were non-randomized.^9–13^ The main characteristics of the non-randomized studies are summarized in Table 1, while those of the randomized trials for both drugs are presented in Tables 2 and 3. Notably, all randomized studies were registrational trials, which are typically designed primarily for regulatory approval rather than to address clinical questions about optimal drug use.

**Table 1.** Characteristics of non-randomized studies evaluating ceftaroline

Study identification	Population			Comparator	Adjustment variables	Clinical cure definition	Risk of bias
	Syndrome	Inclusion criteria	Exclusion criteria				
Arshad *et al*. 2016^9^	HAP	Adults ≥ 18 with HAP or HCAP caused by MRSA	Antimicrobial therapy with an antibiotic of interest in the previous 48 h Incomplete medical records Unvaluable outcome at day 28	Vancomycin Linezolid	ICU setting Cancer Liver disease COPD	Complete resolution of signs and symptoms of pneumonia infection at 28 days from onset of infection	Serious
Athans *et al*. 2016^10^	Osteoarticular infections	Patients with osteomyelitis, septic arthritis, or PJI who received ceftaroline or vancomycin in OPAT as empirical/definitive therapy	< 14 days of OPAT > 1 follow-up assessment Infective endocarditis Concurrent vancomycin + ceftaroline > 24 h Chest or skull bones infection Ceftaroline- or vancomycin-resistant pathogen Receipt of other antibiotics not permitted by the study protocol	Vancomycin	Diabetes MRSA	Any readmission within 180 days of initial hospital discharge where this was due to clinical worsening on therapy, infection recurrence post-therapy or treatment intolerance	Serious
Arshad *et al*. 2017^11^	SAB	Adults ≥ 18 with MRSA bacteraemia susceptible to ceftaroline and with a vancomycin MIC ≥ 1 mg/L	—	Vancomycin Daptomycin	Age Afro-American race *S. aureus* infection in the last year Bone source of BSI COPD	Composite of mortality within 30 days from onset of infection, infection relapse within 42 days, or readmission within 30 days after the end of treatment	Critical
Watkins *et al*. 2018^12^	Spine infections	Adults ≥ 18 with a spine infection who were treated with ceftaroline ≥ 28 days	Ceftaroline dosing information missing Insufficient data to assess treatment response	Vancomycin Daptomycin Ceftriaxone Cefazolin Cefepime Nafcillin	Chronic kidney disease Immunosuppression Cerebral emboli	Resolution of signs and symptoms of infection within 3 months after completion of therapy	Serious
Bitterman *et al*. 2025^13^	SAB	Adults ≥ 18 with at least one positive blood culture growing MRSA who received ceftaroline or vancomycin monotherapy as initial targeted treatment	Antimicrobial therapy > 96 h before starting the study drug	Vancomycin	Age Female sex Charlson index Severe cognitive impairment Nursing home stay Primary bacteraemia Severe sepsis SOFA score PET/TC performed	Composite of mortality at 90 days, persistence of MRSA bacteraemia beyond 7 days or isolation of MRSA from a sterile site beyond 14 days	Moderate

BSI, bloodstream infection; COPD, chronic obstructive pulmonary disease; HAP, hospital-acquired pneumonia; HCAP, healthcare-associated pneumonia; OPAT, outpatient parenteral antimicrobial therapy; PJI, prosthetic joint infection; SAB, *Staphylococcus aureus* bacteraemia

**Table 2.** Ceftaroline randomized studies

Study identification	Population			Comparator	*S. aureus* subgroup (*n*/*N*)	Clinical cure definition	Risk of bias
	Syndrome	Inclusion criteria	Exclusion criteria				
Talbot *et al*. 2007^14^	ABSSSI	Adults ≥ 18 with ABSSSI requiring hospitalization and treatment with IV antimicrobial	Hypersensitivity to any β-lactam or vancomycin Antimicrobial therapy in the previous 96 h Suspected *P. aeruginosa* or anaerobic pathogens Ischaemic ulcer due to PAD Decubitus ulcer Diabetic foot ulcer for more than 7 days Third-degree burn Necrotizing fascitis AIDS Any life-threatining organ disease	Vancomycin + aztreonam	47/100	Resolution of all signs and symptoms of the infection or improvement of the infection such that no further antimicrobial therapy was necessary at 7/14 days after the end of treatment	Low
Ralph Corey *et al*. 2010^15^	ABSSSI	Adults ≥ 18 with ABSSSI that warranted ≥5 days of IV antibacterial therapy	CrCl ≤ 30 mL/min > 24 h of antimicrobial therapy in the previous 96 h Evidence of vancomycin- or aztreonam-resistant pathogens Osteomyelitis/septic arthritis Necrotizing fasciitis Human/animal bite Diabetic foot ulcer Decubitus ulcer Gangrene Burns covering > 5% of the body Mediastinitis Required surgical intervention that could not be performed within 48 h after initiation of therapy	Vancomycin + aztreonam	399/702	Resolution of all signs and symptoms of the infection or improvement of the infection such that no further antimicrobial therapy was necessary at 8/15 days after the end of treatment	Low
Wilcox *et al*. 2010^16^	ABSSSI	Adults ≥ 18 with ABSSSI that warranted ≥5 days of IV antibacterial therapy	CrCl ≤ 30 mL/min > 24 h of antimicrobial therapy in the previous 96 h Evidence of vancomycin- or aztreonam-resistant pathogens Osteomyelitis/septic arthritis Necrotizing fasciitis Human/animal bite Diabetic foot ulcer Decubitus ulcer Gangrene Burns covering > 5% of the body Mediastinitis Required surgical intervention that could not be performed within 48 h after initiation of therapy	Vancomycin + aztreonam	378/694	Resolution of all signs and symptoms of the infection or improvement of the infection such that no further antimicrobial therapy was necessary at 8/15 days after the end of treatment	Low
Low *et al*. 2011^18^	CAP	Adults ≥ 18 with CAP requiring hospitalization and treatment with an IV antimicrobial	PORT classification I, II or V MRSA risk Suspect of atypical pneumonia Antimicrobial therapy in the previous 96 h > 40 mg prednisone/day CrCl < 30 mL/min Significant hepatic disease Neutropenia/thrombocytopenia AIDS	Ceftriaxone	31/627	Resolution of all signs and symptoms of pneumonia or improvement of the infection such that no further antimicrobial therapy was necessary at 8/15 days after the end of treatment	Low
File Jr *et al*. 2011^17^	CAP	Adults ≥ 18 with CAP requiring hospitalization and treatment with an IV antimicrobial	PORT classification I, II or V MRSA risk Suspect of atypical pneumonia Antimicrobial therapy in the previous 96 h > 40 mg prednisone/day CrCl < 30 mL/min Significant hepatic disease Neutropenia/thrombocytopenia AIDS	Ceftriaxone (both group receive additional clarithromycin)	24/591	Resolution of all signs and symptoms of pneumonia or improvement of the infection such that no further antimicrobial therapy was necessary at 8/15 days after the end of treatment	Low
Dryden *et al*. 2016^19^	ABSSSI	Adults ≥ 18 with ABSSSI of sufficient severity to warrant hospitalization and ≥5 days of parenteral antibacterial therapy one or more signs of systemic inflammatory response Underlying comorbidities associated with impaired immune response	Uncomplicated ABSSSI Diabetic foot infection Decubitus ulcer Ulcer due to PAD Necrotizing skin infection Sternal wound infection Body weight > 130 Kg	Vancomycin + aztreonam	191/761	Resolution of all signs and symptoms of the infection or improvement of the infection such that no further antimicrobial therapy was necessary at 8/15 days after the end of treatment	Low

ABSSSI, acute bacterial skin and skin structure infection; CAP, community-acquired pneumonia; PAD, peripheral artery disease; PORT, also known as pneumonia severity index.

**Table 3.** Ceftobiprole randomized studies

Study identification	Population			Comparator	*S. aureus* subgroup (*n*/*N*)	Clinical cure definition	Risk of bias
	Syndrome	Inclusion criteria	Exclusion criteria				
Noel *et al*. 2008 (1)*^20^	ABSSSI	Adults ≥ 18 with complicated ABSSSI caused by documented or suspected Gram-positive bacteria	History of allergic reaction to either cephalosporins or vancomycin CrCl < 30 mL/min Hepatic dysfunction Pregnant or lactating women Neutropenia HIV-infected subjects with CD4 counts < 0.2 × 10^9^/L Diabetic foot infection Osteomyelitis Human/animal bite Antimicrobial therapy > 24 h in the previous 7 days	Vancomycin	359/443	Resolution of all signs and symptoms of the infection or improvement of the infection such that no further antimicrobial therapy was necessary at 7/14 days after the end of treatment	Low
Noel *et al*. 2008 (2)*^21^	ABSSSI	Adults ≥ 18 with complicated ABSSSI	Foreign body infection Osteomyelitis Critical limb ischaemia Septic arthritis	Vancomycin + ceftazidime	375/709	Resolution of all signs and symptoms of infection or improvement to an extent that no further antimicrobial therapy was necessary after 5 days of treatment at 7/14 days after the end of treatment	Low
Nicholson *et al*. 2012^22^	CAP	Adults ≥ 18 with CAP requiring hospitalization and treatment with IV antimicrobial ≥ 3 days	Antimicrobial therapy > 24 h in the previous 3 days Suspected or confirmed pneumonia due to aspiration, atypical bacteria, virus, or *P. jirovecii*	Ceftriaxone + linezolid	13/638	Resolution of signs and symptoms of infection or sufficient improvement such that no further antibacterial therapy was necessary and improvement or no adverse changes in findings on the chest radiograph at 7/14 days after the end of treatment	Low
Awad *et al*. 2014^23^	HAP	Adults ≥ 18 with a diagnosis of pneumonia after ≥ 72 h of hospitalization or stay in a chronic care facility	CrCl < 30 mL/min Hepatic dysfunction Infection with ceftazidime- or ceftobiprole-resistant pathogens Clinical conditions that would interfere with efficacy assessment, such as sustained shock, active tuberculosis, lung abscess, or post-obstructive pneumonia Antimicrobial therapy > 24 h in the previous 48 h	Ceftazidime + linezolid	141/781	Resolution of all signs and symptoms of the infection or improvement of the infection such that no further antimicrobial therapy was necessary at 7/14 days after the end of treatment	Low
Holland *et al*. 2023^3^	SAB	Adults ≥ 18 with complicated SAB confirmed by at least one positive blood culture obtained within 72 h before randomization	Unremovable endovascular prosthetic material Pneumonia Potentially effective antimicrobial therapy > 48 h in the previous 7 days in the absence of persistent SAB	Daptomycin + optional aztreonam	387/387	70-day composite of survival, symptom improvement, SAB clearance, absence of new SAB–related complications, and no use of other potentially effective antibiotics	Low

^*^Noel *et al*. 2008 (1)^20^ and Noel *et al*. 2008 (2)^21^ are two different trials.

ABSSSI, acute bacterial skin and skin structure infection; CAP, community-acquired pneumonia; SAB, *Staphylococcus aureus* bacteraemia.

Among the non-randomized studies, two investigated ceftaroline for osteoarticular and spinal infections,^10,12^ two focused on *S. aureus* bacteraemia,^11,13^ and one addressed hospital-acquired pneumonia (HAP).^9^ Most studies compared ceftaroline with vancomycin, linezolid, or daptomycin, while other β-lactams served as comparators in a minority of cases (Table 1).

All randomized ceftaroline studies were registrational trials. Specifically, four enrolled patients with acute bacterial skin and skin structure infections,^14–16,19^ and two enrolled patients with community-acquired pneumonia (CAP).^17,18^ Two studies on skin infections shared identical protocols despite being conducted at different centres, and the same applied to the two studies on CAP. In all studies on skin infections, ceftaroline monotherapy was compared with vancomycin plus aztreonam, whereas in the studies on CAP, the comparator was ceftriaxone,^18^ with one study adding clarithromycin to both treatment arms.^17^

Regarding ceftobiprole, four of the five trials were registrational: two focused on acute bacterial skin and skin structure infections,^20,21^ one on CAP,^22^ and one on HAP.^23^ The remaining trial was an investigator-initiated, manufacturer-sponsored study enrolling patients with *S. aureus* bacteraemia.^3^ Comparators included vancomycin or vancomycin plus ceftazidime for skin infections, and ceftriaxone plus linezolid for pneumonia. In the bacteraemia trial, the control arm consisted of daptomycin plus aztreonam. All trials reported data on clinical cure.

### Risk of bias

Risk of bias assessments is reported in Supplemental Material (Tables S2–S4). Regarding non-randomized studies, one was deemed to have critical risk of bias, three serious, and one moderate. All randomized studies were deemed to have low risk of bias for the outcome of interest.

### Primary outcome analysis for clinical cure

**Figure 1.**
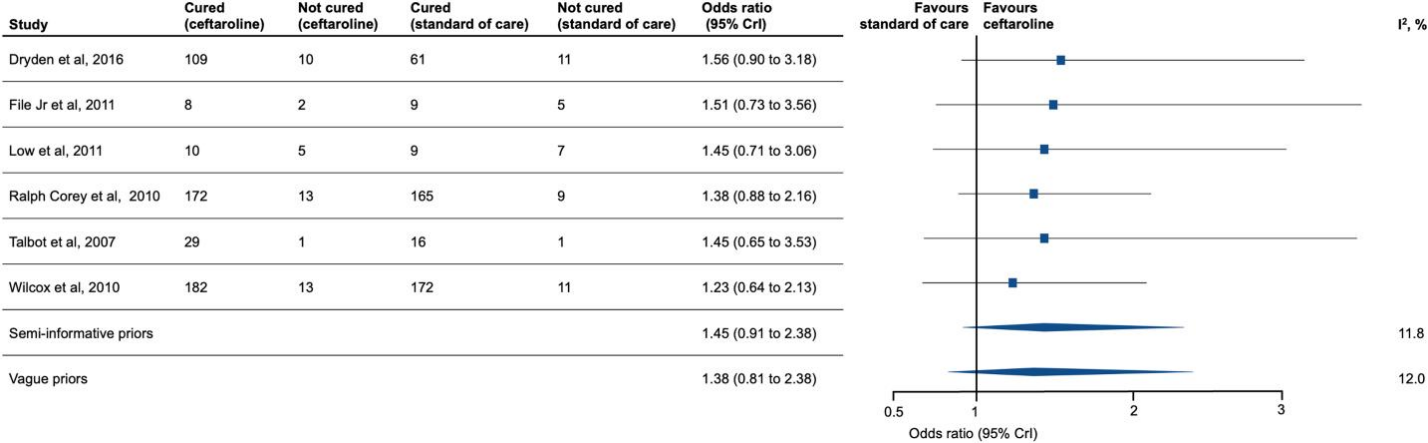
Forest plot of ceftaroline for clinical cure across all infections.

**Figure 2.**
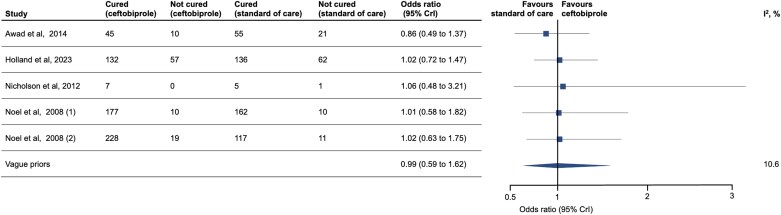
Forest plot of ceftobiprole for clinical cure across all infections.

All trials contributed to the primary outcome analysis for both ceftaroline and ceftobiprole. Apart from the ceftobiprole bacteraemia trial, which used a 70-day composite survival endpoint, the definition of clinical cure was largely consistent across the remaining studies and was defined as resolution of all signs and symptoms of infection or clinical improvement assessed 7 or 14 days after the end of therapy. The primary analysis was a Bayesian random-effects meta-analysis restricted to randomized trials using a vague prior. For ceftaroline, the pooled odds ratio for clinical cure was 1.38 [95% CrI, 0.81–2.44; *τ* = 0.30 (95% CrI, 0.01–0.97); *I*^2^ = 12.0%] (Figure 1). For ceftobiprole, the pooled odds ratio for clinical cure was 0.99 [95% CrI, 0.59–1.62; *τ* = 0.29 (95% CrI, 0.01–0.99); *I*^2^ = 10.6%] (Figure 2).

The funnel plots showed some asymmetry, with smaller studies tending to report larger effect sizes (Figure S2). Given the limited number of included studies, the plots cannot reliably distinguish between publication bias and chance or between-study heterogeneity.

### Exploratory analysis using observationally informed priors

For ceftaroline, we conducted an exploratory analysis using a prior derived from non-randomized studies. This analysis yielded a pooled odds ratio for clinical cure of 1.45 [95% CrI, 0.91–2.38; *τ* = 0.30 (95% CrI, 0.01–0.94); *I*^2^ = 11.8%] (Figure 1). The estimate was similar in direction to the primary analysis, although the use of observationally informed prior should be interpreted cautiously because most contributing non-randomized studies were judged to be at serious or critical risk of bias. Using more conservative discounting factors yielded estimates that remained close to both the primary analysis and the original observationally informed analysis. The pooled odds ratio was 1.41 (95% CrI, 0.87–2.32) when the standard error was inflated by a factor of 2 and 1.41 (95% CrI, 0.85–2.36) when inflated by a factor of 3.

### MRSA exploratory subgroup analysis

**Figure 3.**
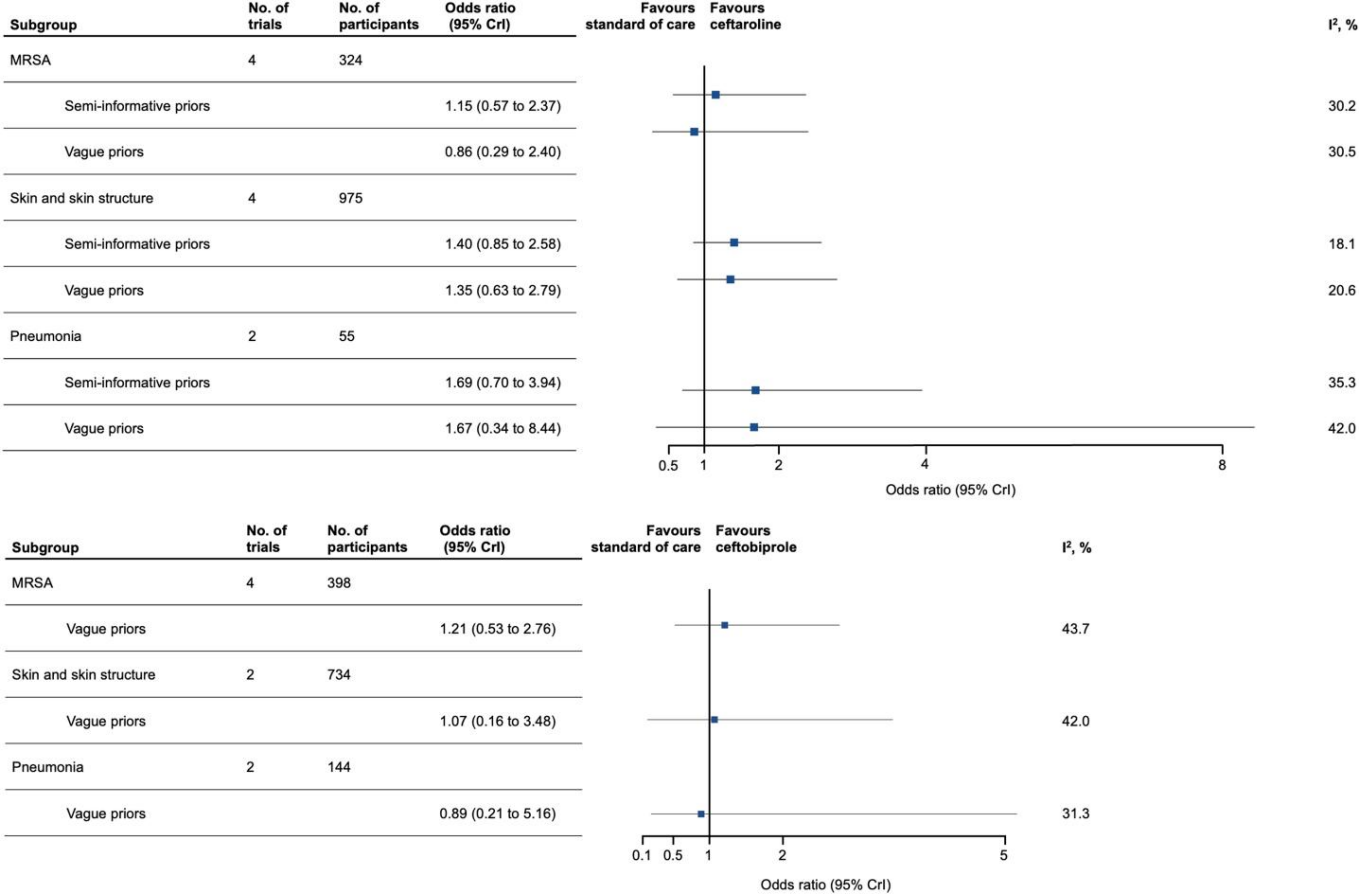
Forest plots for clinical cure in different subgroups.

The MRSA subgroup analysis for ceftaroline was based exclusively on trials of skin and skin structure infections, which were the only studies reporting this subgroup.^14–16,19^ The Bayesian meta-analysis using a semi-informative prior yielded an OR of 1.15 [95% CrI, 0.57–2.37; *τ* = 0.46 (95% CrI, 0.02–1.58); *I*^2^ = 14.6%], whereas the analysis using a vague informative prior yielded an OR of 0.86 [95% CrI, 0.29–2.40; *τ* = 0.50 (95% CrI, 0.02–1.71); *I*^2^ = 17.0%]. The same subgroup was also evaluated in ceftobiprole trials, with four studies contributing to the analysis.^3,20,21,23^ In this analysis, one study was excluded because it had a zero-cell leading to an undefined odds ratio. Using a vague informative prior, the Bayesian meta-analysis resulted in an OR of 1.21 [95% CrI, 0.53–2.76; *τ* = 0.51 (95% CrI, 0.02–1.64); *I*^2^ = 43.7%]. The forest plot for this subgroup is illustrated in Figure 3.

### Acute bacterial skin and skin structure and pulmonary infections exploratory subgroup analysis

Regarding ceftaroline, the skin and skin structure infection analysis using a semi-informative prior resulted in an OR of 1.40 [95% CrI, 0.85–2.58; *τ* = 0.38 (95% CrI, 0.02–1.29); *I*^2^ = 18.1%]. Using a vague prior for the same analysis yielded an OR of 1.35 [95% CrI, 0.63–2.79; *τ* = 0.41 (95% CrI, 0.01–1.46); *I*^2^ = 20.6%]. In the same infection subgroup, the analysis for ceftobiprole using a vague prior resulted in an OR for clinical cure of 1.07 [95% CrI, 0.16–3.48; *τ* = 0.62 (95% CrI, 0.02–2.42); *I*^2^ = 67.8%].

In the pulmonary infection subgroup, the semi-informative prior for ceftaroline resulted in an OR of 1.69 [95% CrI, 0.70–3.94; *τ* = 0.64 (95% CrI, 0.02–2.38); *I*^2^ = 35.3%], whereas the vague prior yielded an OR of 1.67 [95% CrI, 0.34–8.44; *τ* = 0.73 (95% CrI, 0.03–2.91); *I*^2^ = 42.0%]. In contrast, the ceftobiprole analysis yielded an OR of 0.89 [95% CrI, 0.21–5.16; *τ* = 0.84 (95% CrI, 0.03–2.97); *I*^2^ = 31.3%]. The forest plots for these subgroups are illustrated in Figure 3.

### Additional outcomes analysis

In the additional outcomes analysis, safety outcomes were evaluated using vague priors only. For ceftaroline, the analysis of overall adverse events yielded an OR of 0.94 [95% CrI, 0.76–1.14; *τ* = 0.14 (95% CrI, 0.00–0.43); *I*^2^ = 25.7%], while serious adverse events showed an OR of 0.98 [95% CrI, 0.67–1.41; *τ* = 0.21 (95% CrI, 0.01–0.70); *I*^2^ = 19.2%]. For ceftobiprole, overall adverse events were associated with an OR of 1.13 [95% CrI, 0.82–1.50; *τ* = 0.21 (95% CrI, 0.01–0.70); *I*^2^ = 60.5%], whereas serious adverse events yielded an OR of 1.41 [95% CrI, 0.54–3.70; *τ* = 0.68 (95% CrI, 0.06–2.11); *I*^2^ = 79.6%]. The serious adverse event analysis for ceftobiprole showed substantial heterogeneity and wide credible intervals; therefore, this estimate should be interpreted cautiously.

### Frequentist-based sensitivity analysis

To assess the robustness of the Bayesian analysis, we performed a frequentist random-effects meta-analysis. The direction and magnitude of the pooled estimates were unchanged, and interval estimates substantially overlapped, supporting the findings of the main analysis (see Figures S3–S5).

### Certainty of evidence assessment

For ceftaroline, the certainty of evidence for clinical cure in non-urinary tract *S. aureus* infections was rated as low, due to serious risk of bias (subgroup-based analyses) and imprecision. The certainty of evidence across other ceftaroline subgroups ranged from low to very low.

For ceftobiprole, the certainty of evidence for non-urinary tract *S. aureus* infections was moderate, downgraded for imprecision. Although most data were derived from subgroup analyses, one large randomized controlled trial specifically enrolled patients with *S. aureus* bacteraemia. Certainty across the remaining ceftobiprole subgroups ranged from low to very low, primarily due to imprecision. Detailed Summary of Findings tables are provided in Tables S5 and S6.

## Discussion

In this review, we were not able to demonstrate either superiority or inferiority of ceftaroline or ceftobiprole compared with standard-of-care therapies. The credible intervals were overly wide to allow conclusions regarding their efficacy for clinical cure. Accordingly, we found low certainty of evidence supporting the use of ceftaroline for *S. aureus* infections, including MRSA infections. Conversely, the certainty of evidence supporting ceftobiprole for the same infections was rated as moderate. These findings were primarily driven by substantial imprecision, with credible intervals crossing the null value and, in some cases, spanning estimates compatible with both clinically relevant benefit and harm. Moreover, the reliance on subgroup data from trials not specifically designed for *S. aureus* infections further increased uncertainty. Therefore, the pooled estimates should be interpreted as a broad summary of the available randomized evidence rather than as definitive evidence of a common treatment effect across clinically distinct infection syndromes. For ceftaroline, current evidence does not demonstrate superiority over standard-of-care therapy for non-urinary *S. aureus* infections, and randomized evidence outside skin and skin structure infections remains limited. Regarding ceftobiprole, the evidence is mainly supported by data from skin and skin structure infections and by one trial specifically enrolling patients with *S. aureus* bacteraemia, while uncertainty remains concerning its role in MRSA-specific subgroups.

In comparison with the available literature, previous reviews have evaluated these drugs either narratively^4^ or through systematic approaches,^24,25^ including one meta-analysis focused on ceftobiprole.^26^ However, most of these studies concentrated on specific syndromes or assessed combination therapy alongside monotherapy, limiting the ability to isolate the independent contribution of the experimental agent. In contrast, our study systematically included only studies in which these antimicrobials were used as monotherapy, thereby isolating their specific effect. Additional strengths include the updated literature search incorporating the most recent evidence and the explicit separation between randomized, non-randomized evidence, and the use of exploratory analyses using observational informed priors.

Nevertheless, this review has several limitations. First, the number of randomized trials was small, and several subgroup analyses included only two to four studies. These analyses cannot reliably characterize between-study heterogeneity or provide definitive subgroup-specific estimates. Accordingly, subgroup analyses should be considered exploratory and hypothesis-generating, and *τ* and *I*^2^ estimates in these settings should be interpreted cautiously. Second, the overall pooled analyses combined clinically distinct infection syndromes, including acute bacterial skin and skin structure infections, pneumonia, and, for ceftobiprole, *S. aureus* bacteraemia. These syndromes differ in baseline prognosis, outcome relevance, comparator regimens, and trial design. Therefore, the overall pooled estimate should not be interpreted as evidence that the treatment effect is necessarily exchangeable across these conditions. Third, nearly all non-randomized studies evaluating ceftaroline were judged to be at serious or critical risk of bias. Fourth, these studies encompassed heterogeneous clinical syndromes raising concerns about the appropriateness of using such data to inform a Bayesian prior for randomized trials conducted in different populations. For this reason, analyses using observationally informed priors were treated as exploratory sensitivity analyses rather than primary evidence. Fifth, randomized trials were restricted to patients with skin and skin structure infections or pneumonia. Therefore, the applicability of these findings is limited to these syndromes, while the prior incorporated information derived from other infection types. Sixth, the primary outcome was clinical cure, consistent with registrational trials. However, the clinical meaning of this endpoint varies substantially across infection syndromes. For acute bacterial skin and skin structure infections, short-term resolution or improvement of signs and symptoms may represent a clinically meaningful endpoint. In contrast, for invasive *S. aureus* infections, such as bacteraemia or osteoarticular infections, a clinical cure assessment performed 7–14 days after the end of therapy may not adequately capture treatment benefit. In these settings, mortality, relapse, recurrent bacteraemia, and microbiological eradication may be more informative. The ceftobiprole trial by Holland *et al*.^3^ represents the main exception among the randomized evidence.^3^ Seventh, the funnel plots showed some asymmetry, although the small number of included studies prevents any reliable distinction between chance, clinical heterogeneity, small-study effects, or publication bias. This issue is particularly relevant for ceftaroline, for which the point estimates tended to favour the experimental drug but remained imprecise, with credible intervals crossing the null. Because the randomized evidence for ceftaroline was derived from registrational industry-sponsored trials, optimism or sponsorship bias cannot be excluded^27^ Therefore, the apparent favourable direction of effect for ceftaroline should be interpreted cautiously and should not be considered evidence of superiority over standard-of-care therapy. Eighth, the heterogeneity in comparators further compounds the interpretation of the results, because the pooled estimates do not represent comparison against a single standard-of-care agent.

Overall, this study highlights the persistent uncertainty surrounding these cephalosporins in clinical practice. Despite their pharmacological advantages and activity against MRSA, robust evidence demonstrating clinically meaningful superiority or equivalence over established therapies remains lacking. The absence of clear benefit should not be interpreted as proof of equivalence, but rather as evidence of insufficient precision and limited syndrome-specific randomized data. Additionally, our findings underscore the gap between regulatory approval trials and the clinical scenarios in which these agents are frequently used. Many real-world applications involve severe or invasive *S. aureus* infections, although high-quality randomized data in these settings remain scarce. From a clinical perspective, our findings support a cautious and indication-specific use of ceftaroline and ceftobiprole. For ceftaroline, the available randomized evidence is mainly derived from skin and skin structure infection and pneumonia trials, with MRSA-specific data limited to skin and skin structure infections. The lack of randomized evidence for MRSA bacteraemia or other invasive MRSA infections should be interpreted as an evidence gap rather than as evidence of lack of efficacy. For ceftobiprole, moderate-certainty evidence in bacteraemia suggests a potential role, although uncertainty persists in MRSA-specific subgroups. These results do not justify routine preference of these agents over established standard-of-care therapies based on presumed superiority. Instead, their use may be individualized, considering patient characteristics, intolerance to other agents, local resistance patterns, and antimicrobial stewardship principles. From a policy standpoint, our analysis underscores the importance of generating syndrome-specific evidence before widespread adoption of newer, often more costly agents.

Future research should include adequately powered randomized controlled trials specifically enrolling patients with *S. aureus* infections, particularly MRSA. In doing this, future trials should incorporate clinically meaningful endpoints such as mortality, relapse, microbiological eradication, and patient-centred outcomes. Until such evidence is available, pooled estimates across heterogeneous syndromes should be interpreted as a map of the current evidence base rather than as definitive guidance for all *S. aureus* infections.

## Supplementary Material

dkag244_Supplementary_Data

## Data Availability

The data underlying this article will be shared on reasonable request to the corresponding author.

## Supplementary data

Figures S1 to S2 and Tables S2 to S4, S1 and S5 to S6 are available as Supplementary data at *JAC* Online.
