# Supplementary material for: Ceftaroline and ceftobiprole monotherapy for the treatment of Staphylococcus aureus infections: a systematic review and Bayesian meta-analysis
Source: J Antimicrob Chemother. 2026 Jul 14;81(8):dkag244. doi: 10.1093/jac/dkag244 (PMC13366531; doi:10.1093/jac/dkag244)

Table S1. Search string

**PubMed**
"ceftaroline fosamil" [Supplementary Concept] OR ceftaroline[tw] OR "T 91825" [Supplementary Concept] OR “T 91825”[tw] OR T91825[tw] OR “T-91825”[tw] OR zinforo[tw] OR teflaro[tw] OR “TAK-599”[tw] OR TAK599[tw] OR “PPI-0903”[tw] OR PPI0903[tw] OR "ceftobiprole medocaril" [Supplementary Concept] OR ceftobiprole[tw] OR "ceftobiprole" [Supplementary Concept] OR BAL5788[tw] OR “BAL 5788”[tw] OR “BAL-5788”[tw] OR zevtera[tw] OR mabelio[tw] OR BAL9149[tw] OR “BAL 9149”[tw] OR “BAL-9149”[tw] AND "Methicillin-Resistant Staphylococcus aureus"[Mesh] OR MRSA[tw] OR “Methicillin-Resistant Staphylococcus aureus”[tw] OR "Staphylococcus aureus"[Mesh] OR “Staphylococcus aureus”[tw] OR “S. aureus”[tw] OR “aureus”[tw] OR "Staphylococcus"[Mesh] OR Staphylococcus[tw] OR “Gram positive”[tw] OR “Gram-positive”[tw] OR "Gram-Positive Endospore-Forming Rods"[Mesh] OR cocci[tw]
**Web of Science**
"ceftaroline fosamil" OR ceftaroline OR "T 91825" OR "T 91825" OR T91825 OR T-91825 OR zinforo OR teflaro OR TAK-599 OR TAK599 OR PPI-0903 OR PPI0903 OR "ceftobiprole medocaril" OR ceftobiprole OR ceftobiprole OR BAL5788 OR "BAL 5788" OR BAL-5788 OR zevtera OR mabelio OR BAL9149 OR "BAL 9149" OR BAL-9149 AND "Methicillin-Resistant Staphylococcus aureus" OR MRSA OR "Methicillin-Resistant Staphylococcus aureus" OR "Staphylococcus aureus" OR "Staphylococcus aureus" OR "S. aureus" OR aureus OR Staphylococcus OR Staphylococcus OR "Gram positive" OR Gram-positive OR "Gram-Positive Endospore-Forming Rods" OR cocci
**Embase**
'ceftaroline fosamil':tn OR ceftaroline OR 'T 91825':tn OR 'T 91825' OR T91825 OR T-91825 OR zinforo OR teflaro OR TAK-599 OR TAK599 OR PPI-0903 OR PPI0903 OR 'ceftobiprole medocaril':tn OR ceftobiprole OR ceftobiprole:tn OR BAL5788 OR 'BAL 5788' OR BAL-5788 OR zevtera OR mabelio OR BAL9149 OR 'BAL 9149' OR BAL-9149 AND 'Methicillin-Resistant Staphylococcus aureus'/exp OR MRSA OR

'Methicillin-Resistant Staphylococcus aureus' OR 'Staphylococcus aureus'/exp OR 'Staphylococcus aureus' OR 'S. aureus' OR aureus OR Staphylococcus/exp OR Staphylococcus OR 'Gram positive' OR Gram-positive OR 'Gram-Positive Endospore-Forming Rods'/exp OR cocci
**Scopus**
CHEM(term) OR TITLE-ABS-KEY(ceftaroline) OR CHEM(term) OR TITLE-ABS-KEY("T 91825") OR TITLE-ABS-KEY(T91825) OR TITLE-ABS-KEY(T-91825) OR TITLE-ABS-KEY(zinforo) OR TITLE-ABS-KEY(teflaro) OR TITLE-ABS-KEY(TAK-599) OR TITLE-ABS-KEY(TAK599) OR TITLE-ABS-KEY(PPI-0903) OR TITLE-ABS-KEY(PPI0903) OR CHEM(term) OR TITLE-ABS-KEY(ceftobiprole) OR CHEM(term) OR TITLE-ABS-KEY(BAL5788) OR TITLE-ABS-KEY("BAL 5788") OR TITLE-ABS-KEY(BAL-5788) OR TITLE-ABS-KEY(zevtera) OR TITLE-ABS-KEY(mabelio) OR TITLE-ABS-KEY(BAL9149) OR TITLE-ABS-KEY("BAL 9149") OR TITLE-ABS-KEY(BAL-9149) AND INDEXTERMS("Methicillin-Resistant Staphylococcus aureus") OR TITLE-ABS-KEY(MRSA) OR TITLE-ABS-KEY("Methicillin-Resistant Staphylococcus aureus") OR INDEXTERMS("Staphylococcus aureus") OR TITLE-ABS-KEY("Staphylococcus aureus") OR TITLE-ABS-KEY("S. aureus") OR TITLE-ABS-KEY(aureus) OR INDEXTERMS(Staphylococcus) OR TITLE-ABS-KEY(Staphylococcus) OR TITLE-ABS-KEY("Gram positive") OR TITLE-ABS-KEY(Gram-positive) OR INDEXTERMS("Gram-Positive Endospore-Forming Rods") OR TITLE-ABS-KEY(cocci)

Table S2. Risk of bias assessment of non-randomized controlled studies (ceftaroline only)

Risk of bias domains

Study	D1	D2	D3	D4	D5	D6	D7	Overall
Arshad et al, 2016	X	X	-	+	+	-	+	X
Athans et al, 2016	X	!	-	-	+	-	-	!
Arshad et al, 2017	-	X	-	-	+	-	-	X
Watkins et al, 2018	-	X	-	+	-	-	+	X
Bitterman et al, 2025	-	+	+	+	+	-	+	-

Domains:
D1: Bias due to confounding.
D2: Bias due to selection of participants.
D3: Bias in classification of interventions.
D4: Bias due to deviations from intended interventions.
D5: Bias due to missing data.
D6: Bias in measurement of outcomes.
D7: Bias in selection of the reported result.

Judgement
! Critical
X Serious
- Moderate
+ Low

Table S3. Risk of bias assessment of randomized controlled trials for ceftaroline

Study	Risk of bias domains					
	D1	D2	D3	D4	D5	Overall
Talbot et al, 2017	+	+	+	+	+	+
Ralph Corey et al, 2010	+	+	+	+	+	+
Wilcox et al, 2010	+	+	+	+	+	+
Low et al, 2011	+	+	+	+	+	+
File Jr et al, 2011	+	+	+	+	+	+
Dryden et al, 2016	+	+	+	+	+	+

Domains:
D1: Bias arising from the randomization process.
D2: Bias due to deviations from intended intervention.
D3: Bias due to missing outcome data.
D4: Bias in measurement of the outcome.
D5: Bias in selection of the reported result.

Judgement
+ Low

Table S4. Risk of bias assessment of randomized controlled trials for ceftobiprole

Study	D1	D2	D3	D4	D5	Overall
Noel et al, 2008 (1)	+	+	+	+	+	+
Noel et al, 2008 (2)	+	+	+	+	+	+
Nicholson et al, 2012	+	+	+	+	+	+
Awad et al, 2014	+	+	+	+	+	+
Holland et al, 2023	+	+	+	+	+	+

Risk of bias domains

Domains:
D1: Bias arising from the randomization process.
D2: Bias due to deviations from intended intervention.
D3: Bias due to missing outcome data.
D4: Bias in measurement of the outcome.
D5: Bias in selection of the reported result.

Judgement
+ Low

Table S5. GRADE assessments for ceftaroline

Question 1: Ceftaroline compared to standard of care for non–urinary tract *Staphylococcus aureus* infections

Certainty assessment							Nº of patients		Effect		Certainty	Importance
**Nº of studies**	**Study design**	**Risk of bias**	**Inconsistency**	**Indirectness**	**Imprecision**	**Other considerations**	**ceftaroline**	**standard of care**	**Relative (95% CrI)**	**Absolute (95% CI)**		
**Clinical cure**												
6	randomised trials	serious[a]	not serious	not serious	serious[b]	none	510/554 (92.1%)	432/476 (90.8%)	**OR 1.45** (0.91 to 2.38)	**27 more per 1,000** (from 8 fewer to 51 more)	⨁⨁◯◯ Low[a,b]	IMPORTANT

CI: confidence interval; **CrI:** credible interval; **OR:** odds ratio

Explanations

a. Although all included studies were randomized controlled trials, the analyses relied on subgroup populations in which the benefits of randomization cannot be assumed to be preserved.

b. The credible interval for the primary outcome crossed the null value (pooled OR, 1.45 [95% CrI, 0.91–2.38]).

Question 2: Ceftaroline compared to standard of care for *Staphylococcus aureus* pneumonia

Certainty assessment							Nº of patients		Effect		Certainty	Importance
**Nº of studies**	**Study design**	**Risk of bias**	**Inconsistency**	**Indirectness**	**Imprecision**	**Other considerations**	**ceftaroline**	**standard of care**	**Relative (95% CrI)**	**Absolute (95% CI)**		
**Clinical cure**												
2	randomised trials	serious[a]	not serious	not serious	very serious[b]	none	18/25 (72.0%)	18/30 (60.0%)	**OR 1.69** (0.70 to 3.94)	**117 more per 1,000** (from 88 fewer to 255 more)	⨁◯◯◯ Very low[a,b]	IMPORTANT

CI: confidence interval; **CrI:** credible interval**; OR:** odds ratio

Explanations

a. Although all included studies were randomized controlled trials, the analyses relied on subgroup populations in which the benefits of randomization cannot be assumed to be preserved.

b. Imprecision was very serious due to the small subgroup size and a wide credible interval crossing the null and encompassing both clinically important harm and substantial benefit (OR, 1.69 [95% CrI, 0.70–3.94]).

Question 3: Ceftaroline compared to standard of care for *Staphylococcus aureus* skin and skin structure infections

Certainty assessment							№ of patients		Effect		Certainty	Importance
**№ of studies**	**Study design**	**Risk of bias**	**Inconsistency**	**Indirectness**	**Imprecision**	**Other considerations**	**ceftaroline**	**standard of care**	**Relative (95% CrI)**	**Absolute (95% CI)**		
**Clinical cure**												
4	randomised trials	serious[a]	not serious	not serious	serious[b]	none	492/529 (93.0%)	414/446 (92.8%)	**OR 1.40** (0.85 to 2.58)	**19 more per 1,000** (from 12 fewer to 43 more)	⨁⨁◯◯ Low[a,b]	CRITICAL

CI: confidence interval; **CrI:** credible interval; **OR:** odds ratio

Explanations

a. Although all included studies were randomized controlled trials, the analyses relied on subgroup populations in which the benefits of randomization cannot be assumed to be preserved.

b. The estimate was affected by imprecision, with a wide credible interval crossing the null value and encompassing both clinically relevant benefit and harm (OR, 1.40 [95% CrI, 0.85–2.58]).

Question 4: Ceftaroline compared to standard of care for MRSA infections

Certainty assessment							№ of patients		Effect		Certainty	Importance
**№ of studies**	**Study design**	**Risk of bias**	**Inconsistency**	**Indirectness**	**Imprecision**	**Other considerations**	**ceftaroline**	**standard of care**	**Relative (95% CrI)**	**Absolute (95% CI)**		
**Clinical cure**												
4	randomised trials	serious[a]	not serious	very serious[b]	very serious[c]	none	167/182 (91.8%)	132/142 (93.0%)	**OR 1.24** (0.58 to 2.77)	**13 more per 1,000** (from 45 fewer to 44 more)	⨁◯◯◯ Very low[a,b,c]	IMPORTANT

CI: confidence interval; **CrI:** credible interval; **OR:** odds ratio

Explanations

a. Although all included studies were randomized controlled trials, the analyses relied on subgroup populations in which the benefits of randomization cannot be assumed to be preserved.

b. The studies were conducted in patients with skin and skin structure infections. No study specifically evaluated the intervention in other MRSA infections.

c. The estimate was affected by substantial imprecision, with a wide credible interval crossing the null value and encompassing both clinically relevant benefit and harm (OR, 1.24 [95% CrI, 0.58–2.77]).

Table S6. GRADE assessments for ceftobiprole

Question 5: Ceftobiprole compared to standard of care for non-urinary tract *Staphylococcus aureus* infections

Certainty assessment							Nº of patients		Effect		Certainty	Importance
Nº of studies	Study design	Risk of bias	Inconsistency	Indirectness	Imprecision	Other considerations	ceftobiprole	standard of care	Relative (95% CI)	Absolute (95% CI)		
**Clinical cure**												
5	randomised trials	not serious[a]	not serious	not serious	serious[b]	none	589/685 (86.0%)	475/580 (81.9%)	**OR 0.99** (0.59 to 1.62)	**1 fewer per 1,000** (from 92 fewer to 61 more)	⨁⨁⨁◯ Moderate[a,b]	IMPORTANT

CI: confidence interval; **OR:** odds ratio

Explanations

a. The analyses relied on four subgroup populations derived from randomized controlled trials, for which the benefits of randomization may not be fully preserved. However, one large randomized controlled trial specifically enrolled patients with Staphylococcus aureus bacteremia.

b. The estimate was affected by imprecision, with a wide credible interval crossing the null value and encompassing both clinically relevant benefit and harm (OR, 0.99 [95% CrI, 0.59–1.62).

Question 6: Ceftobiprole compared to standard of care for *Staphylococcus aureus* pneumonia

Certainty assessment							№ of patients		Effect		Certainty	Importance
№ of studies	Study design	Risk of bias	Inconsistency	Indirectness	Imprecision	Other considerations	ceftobiprole	standard of care	Relative (95% CI)	Absolute (95% CI)		
**Clinical cure**												
2	randomised trials	serious[a]	not serious	not serious	very serious[b]	none	52/62 (83.9%)	60/82 (73.2%)	**OR 0.89** (0.21 to 5.16)	**23 fewer per 1,000** (from 368 fewer to 202 more)	⨁◯◯◯ Very low[a,b]	IMPORTANT

CI: confidence interval; **OR:** odds ratio

Explanations

a. Although all included studies were randomized controlled trials, the analyses relied on subgroup populations in which the benefits of randomization cannot be assumed to be preserved.

b. Imprecision was very serious due to the small subgroup size and a wide credible interval crossing the null and encompassing both clinically important harm and substantial benefit (OR, 0.89 [95% CrI, 0.21–5.16]).

Question 7: Ceftobiprole compared to standard of care for *Staphylococcus aureus* skin and skin structure infections

Certainty assessment							Nº of patients		Effect		Certainty	Importance
**Nº of studies**	**Study design**	**Risk of bias**	**Inconsistency**	**Indirectness**	**Imprecision**	**Other considerations**	**ceftobiprole**	**standard of care**	**Relative (95% CI)**	**Absolute (95% CI)**		
**Clinical cure**												
2	randomised trials	serious[a]	not serious	not serious	serious[b]	none	405/434 (93.3%)	279/300 (93.0%)	**OR 1.07** (0.16 to 3.48)	**4 more per 1,000** (from 250 fewer to 49 more)	⨁⨁◯◯ Low[a,b]	CRITICAL

CI: confidence interval; **OR:** odds ratio

Explanations

a. Although all included studies were randomized controlled trials, the analyses relied on subgroup populations in which the benefits of randomization cannot be assumed to be preserved.

b. The estimate was affected by substantial imprecision, with a wide credible interval crossing the null value and encompassing both clinically relevant benefit and harm (OR, 1.07 [95% CrI, 0.16–3.48]).

Question 8: Ceftobiprole compared to standard of care for MRSA infections

Certainty assessment							№ of patients		Effect		Certainty	Importance
№ of studies	Study design	Risk of bias	Inconsistency	Indirectness	Imprecision	Other considerations	ceftobiprole	standard of care	Relative (95% CI)	Absolute (95% CI)		
**Clinical cure**												
2	randomised trials	serious[a]	not serious	not serious	very serious[b]	none	187/221 (84.6%)	142/177 (80.2%)	**OR 1.21** (0.53 to 2.76)	**29 more per 1,000** (from 120 fewer to 116 more)	⊕◯◯◯ Very low[a,b]	IMPORTANT

CI: confidence interval; **OR:** odds ratio

Explanations

a. Although all included studies were randomized controlled trials, the analyses relied on subgroup populations in which the benefits of randomization cannot be assumed to be preserved.

b. The estimate was affected by substantial imprecision, with a wide credible interval crossing the null value and encompassing both clinically relevant benefit and harm (OR, 1.21 [95% CrI, 0.53–2.76]).

Figure S1. PRISMA diagram

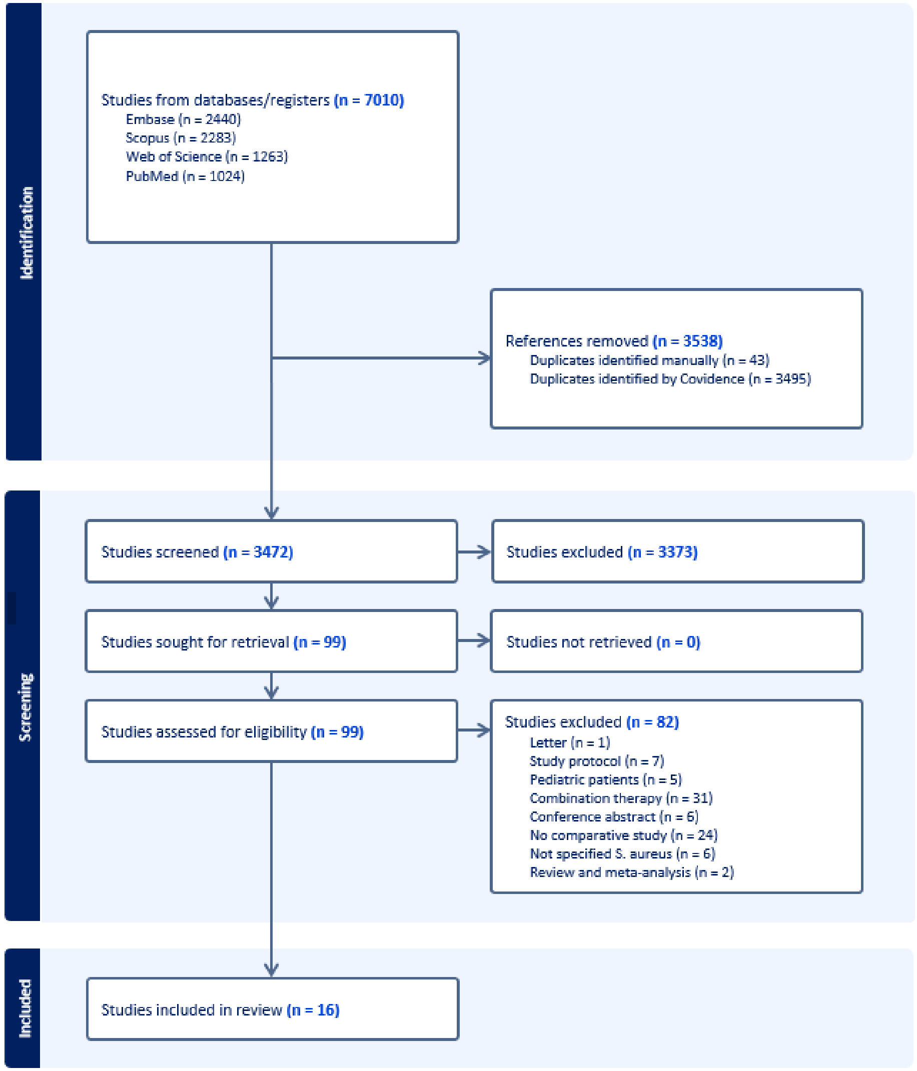

Figure S2. Funnel plots for primary outcome

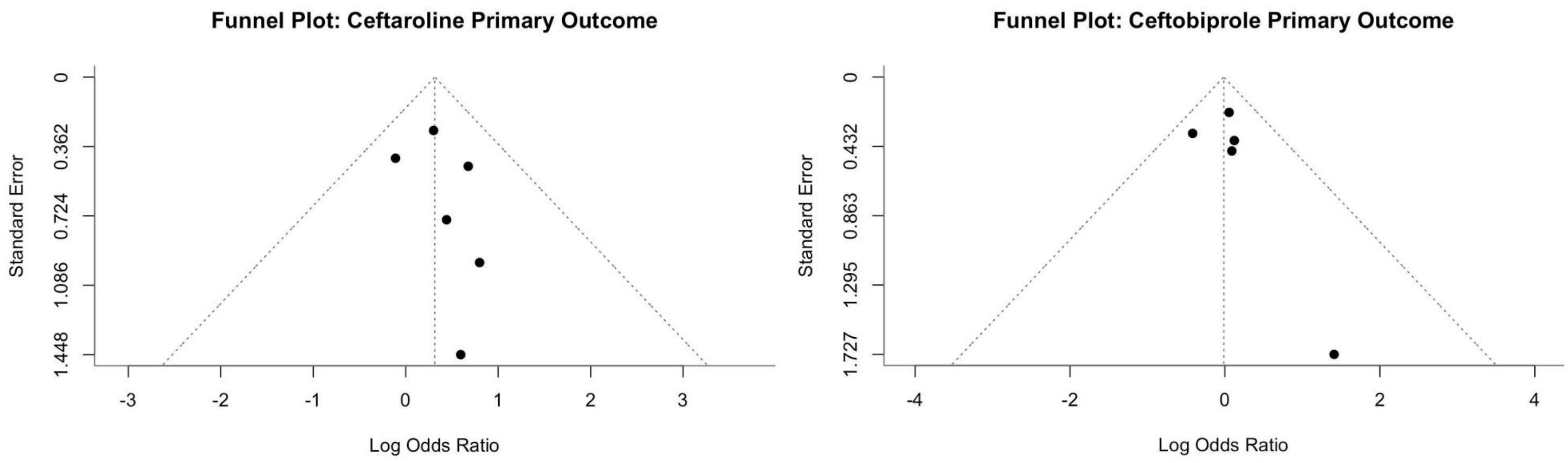

Figure S3. Frequentist meta-analysis of non-randomized studies for ceftaroline

Study	OR	95% CI	OR	95%-CI	Weight
Watkins et al, 2018	0.3988	1.0766	1.49	[0.18; 12.29]	7.0%
Arshad et al, 2016	1.3083	0.9195	3.70	[0.61; 22.43]	9.5%
Arshad et al, 2017	-0.3285	0.6845	0.72	[0.19; 2.75]	17.2%
Athans et al, 2016	0.5247	0.5038	1.69	[0.63; 4.54]	31.8%
Bitterman et al, 2025	0.7129	0.4836	2.04	[0.79; 5.26]	34.5%
**Random-effects model**			**1.66**	**[0.95; 2.90]**	**100.0%**

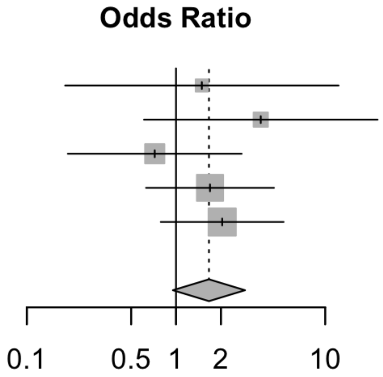

Heterogeneity: $I^2 = 0.0\%$, $\tau^2 = 0$, $p = 0.6550$

Figure S4. Frequentist meta-analysis for ceftaroline

Primary outcome (clinical cure)

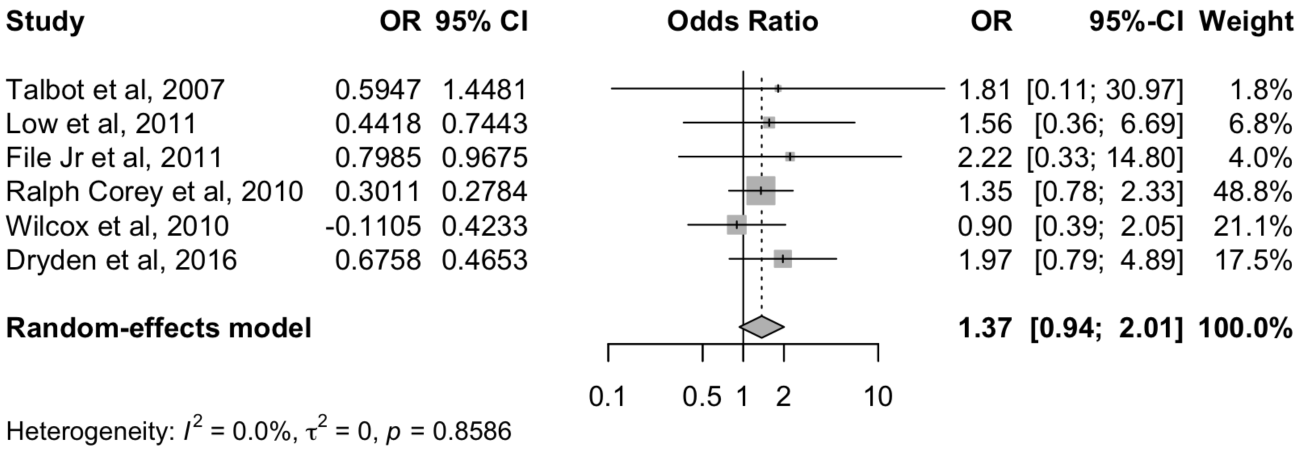

MRSA subgroup

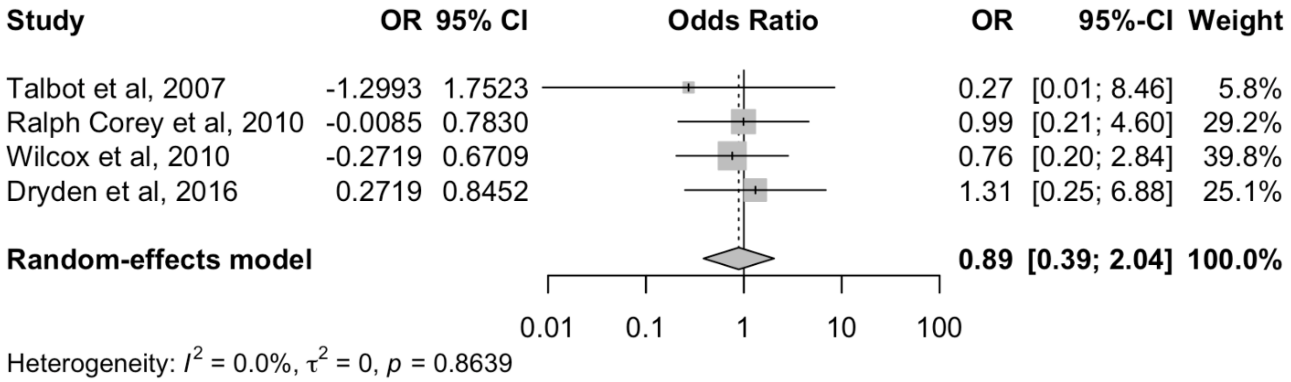

Acute bacterial skin and skin structure infection subgroup

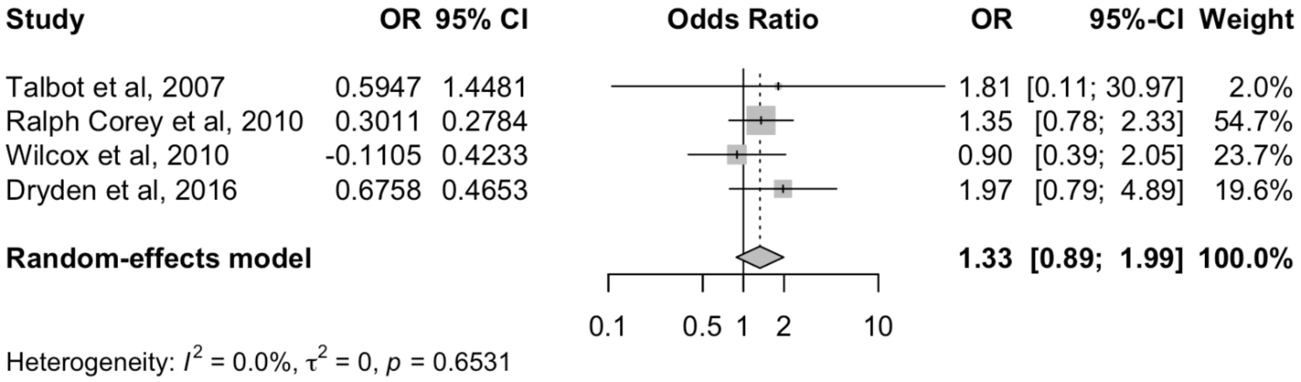

Lung infection subgroup

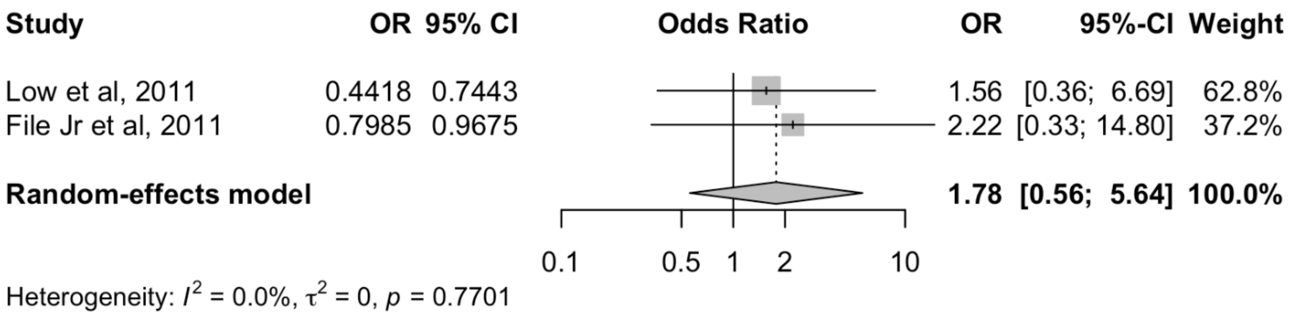

Adverse events analysis

Study	OR	95% CI	OR	95%-CI	Weight
Talbot et al, 2007	-0.6004	0.4395	0.55	[0.23; 1.30]	2.8%
Low et al, 2011	0.2234	0.2068	1.25	[0.83; 1.88]	12.7%
File Jr et al, 2011	-0.1734	0.1648	0.84	[0.61; 1.16]	19.9%
Ralph Corey et al, 2010	-0.0160	0.1679	0.98	[0.71; 1.37]	19.2%
Wilcox et al, 2010	-0.1893	0.1545	0.83	[0.61; 1.12]	22.7%
Dryden et al, 2016	0.0145	0.1542	1.01	[0.75; 1.37]	22.8%
**Random-effects model**			**0.94**	**[0.81; 1.08]**	**100.0%**

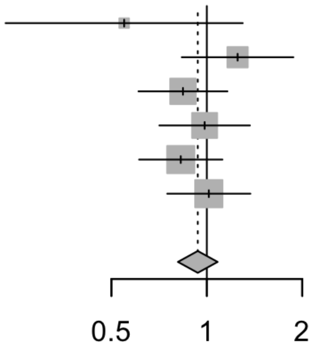

Heterogeneity: $I^2 = 0.0\%$, $\tau^2 < 0.0001$, $p = 0.4330$

Severe adverse events analysis

Study	OR	95% CI	OR	95%-CI	Weight
Low et al, 2011	0.0279	0.2396	1.03	[0.64; 1.64]	37.4%
File Jr et al, 2011	-0.1460	0.2708	0.86	[0.51; 1.47]	29.2%
Ralph Corey et al, 2010	0.2877	0.3896	1.33	[0.62; 2.86]	14.1%
Wilcox et al, 2010	-0.1458	0.3743	0.86	[0.42; 1.80]	15.3%
Dryden et al, 2016	-0.1764	0.7344	0.84	[0.20; 3.54]	4.0%
**Random-effects model**			**0.98**	**[0.73; 1.30]**	**100.0%**

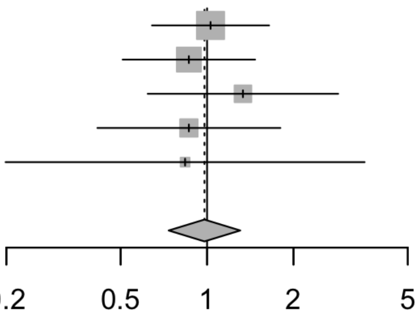

Heterogeneity: $I^2 = 0.0\%$, $\tau^2 = 0$, $p = 0.9040$

Figure S5. Frequentist meta-analysis for ceftobiprole

Primary outcome (clinical cure)

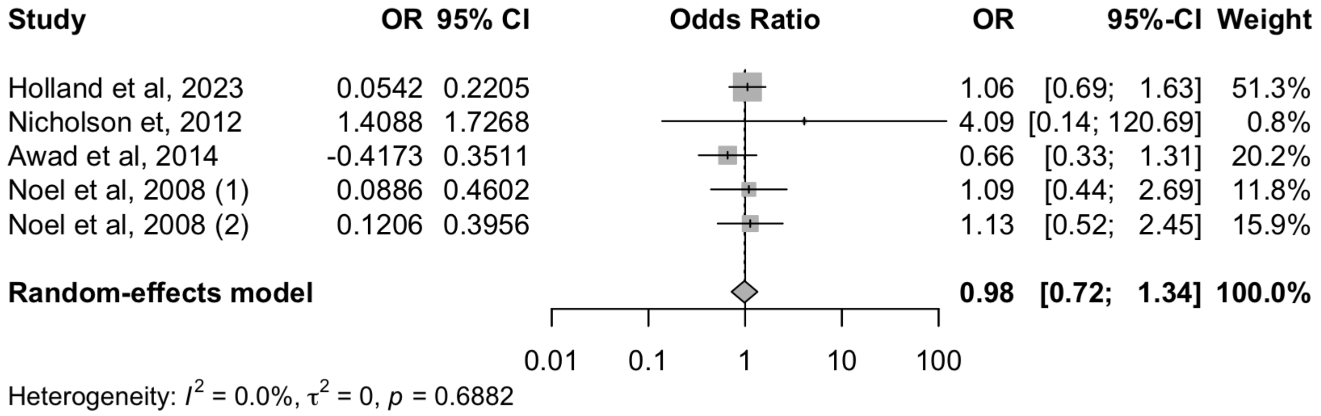

MRSA subgroup

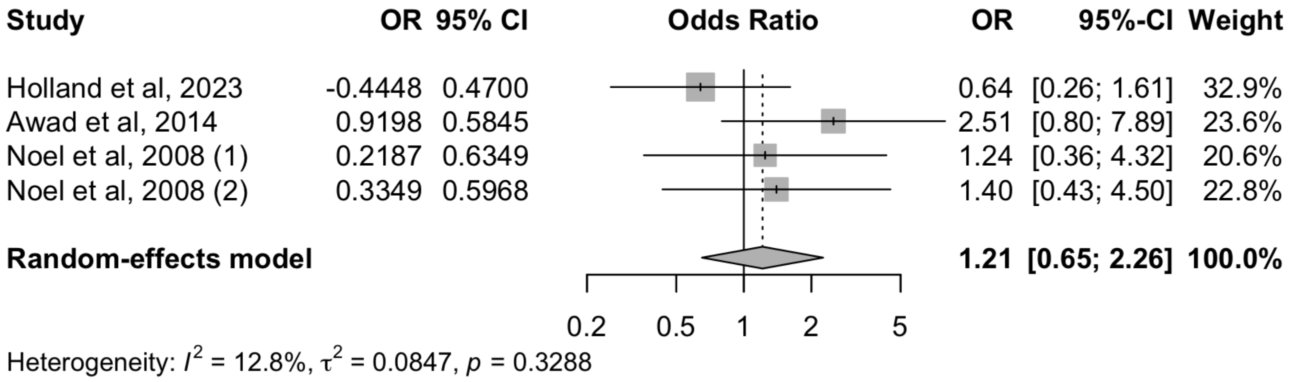

Acute bacterial skin and skin structure infection subgroup

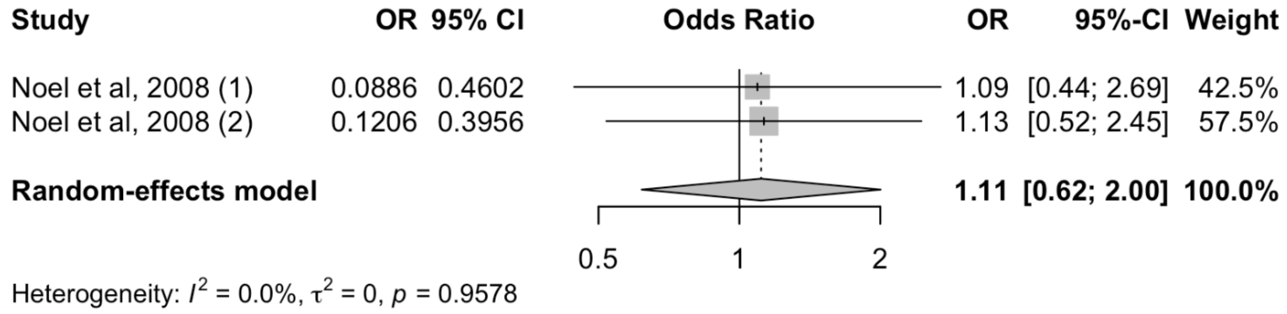

Lung infection subgroup

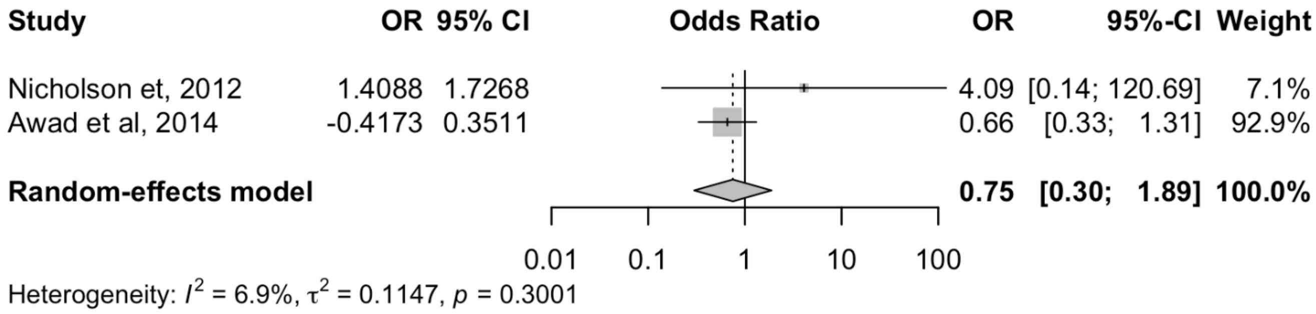

Adverse events analysis

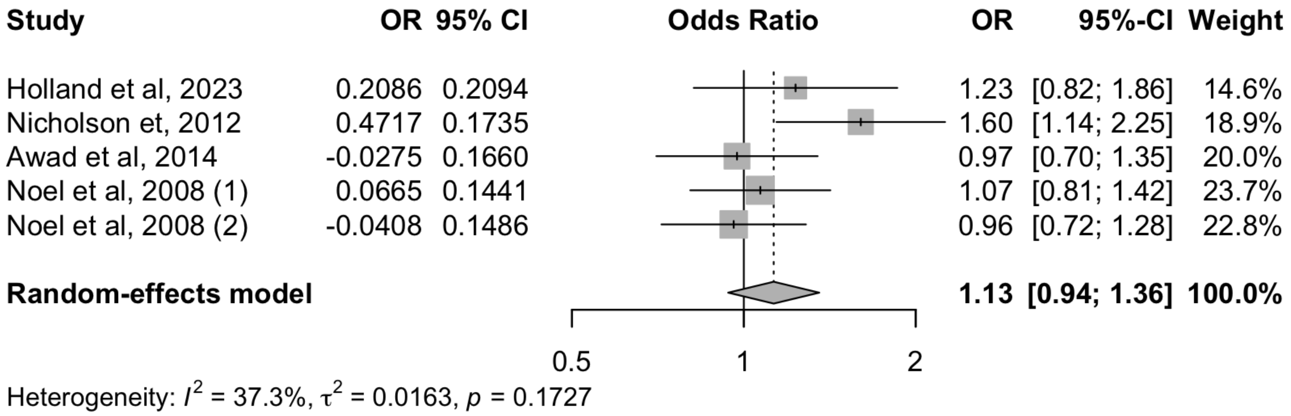

Severe adverse events analysis

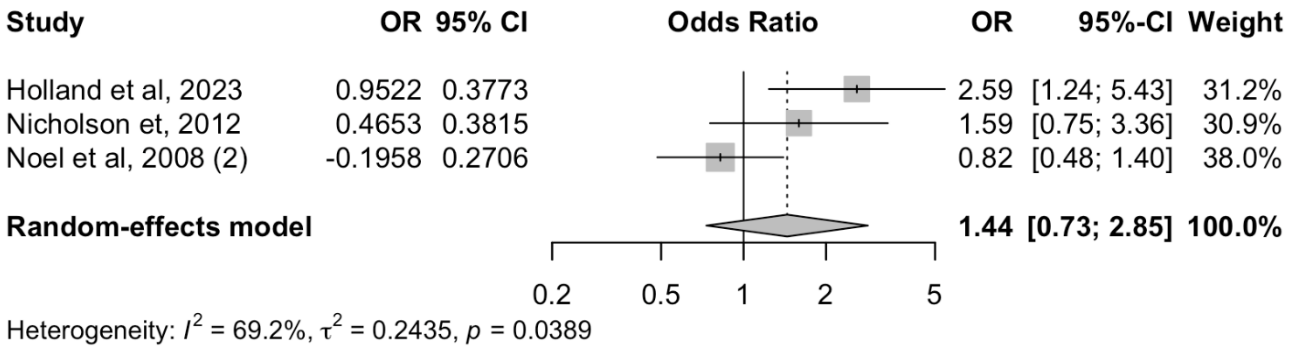

Supplement: dkag244_Supplementary_Data [file dkag244_supplementary_data.zip › Supplemental.pdf]
